# Supplementary material for: Preclinical Long-Term Stability and Forced Degradation Assessment of EPICERTIN, a Mucosal Healing Biotherapeutic for Inflammatory Bowel Disease
Source: Pharmaceutics. 2025 Feb 15;17(2):259. doi: 10.3390/pharmaceutics17020259 (PMC11859197; doi:10.3390/pharmaceutics17020259)
Supplement: Supplementary file 1 [file pharmaceutics-17-00259-s001.zip › pharmaceutics-3481693-supplementary.pdf]

Table S1. EPICERTIN DS 2-year long-term stability assessment results summary: 5 °C condition.

					**Temperature 5 °C**											
**Test Parameter**	**Test Method**	**Acceptance Criteria**	**0 months**		**1 month**		**3 months**		**6 months**		**9 months**		**12 months**		**24 months**	
Appearance	Visible appearance	Clear, colorless liquid, free of visible particles (CCLFVP)	Pass	CCLFVP	Pass	CCLFVP	Pass	CCLFVP	Pass	CCLFVP	Pass	CCLFVP	Pass	CCLFVP	Pass	CCLFVP
Protein Concentration	A_{280}	1 ± 0.2 mg/mL	Pass	1.06 ± 0.004	Pass	1.03 ± 0.010	Pass	1.06 ± 0.019	Pass	1.07 ± 0.005	Pass	1.11 ± 0.013	Pass	1.13 ± 0.047	Pass	1.13 ± 0.025
Physicochemical Properties	pH	7.4 ± 0.2	Pass	7.25	Pass	7.32	Pass	7.36	Pass	7.34	Pass	7.32	Pass	7.34	Pass	7.32
Identity	ESI-MS	12280 ± 3 Da	Pass	12280	Pass	12280	Pass	12281	Pass	12280	Pass	12280	Pass	12280	Pass	12280
Purity	Reducing SDS-PAGE	≥ 95% EPICERTIN	Pass	100.00%	Pass	100.00%	Pass	100.00%	Pass	100.00%	Pass	100.00%	Pass	100.00%	Pass	100.00%
Purity	Size exclusion HPLC	16.4 ± 0.2 min retention time and ≥ 95% AUC	Pass	100.00%	Pass	100.00%	Pass	100.00%	Pass	100.00%	Pass	100.00%	Pass	100.00%	Pass	100.00%
Potency	GM1/KDEL ELISA	EC50 shift < ± 30% of reference standard	Pass	+2.85%	Pass	+2.88%	Pass	+16%	Pass	+4.9%	Pass	-7.7%	Pass	-27.2%	Pass	-9.6%

Table S2. EPICERTIN DS 2-year long-term stability assessment results summary: 25 °C/60% RH condition.

					**Temperature 25 °C/60% RH**											
**Test Parameter**	**Test Method**	**Acceptance Criteria**	**0 months**		**1 month**		**3 months**		**6 months**		**9 months**		**12 months**		**24 months**	
Appearance	Visible appearance	Clear, colorless liquid, free of visible particles (CCLFVP)	Pass	CCLFVP	Pass	CCLFVP	Pass	CCLFVP	Pass	CLFVP	Pass	CCLFVP	Pass	CCLFVP	Pass	CCLFVP
Protein Concentration	A_{280}	1 ± 0.2 mg/mL	Pass	1.06 ± 0.004	Pass	1.05 ± 0.006	Pass	1.06 ± 0.004	Pass	1.12 ± 0.008	Fail	1.22 ± 0.004	Fail	1.26 ± 0.022	Fail	1.35 ± 0.032
Physicochemical Properties	pH	7.4 ± 0.2	Pass	7.25	Pass	7.41	Pass	7.32	Pass	7.34	Pass	7.31	Pass	7.37	Pass	7.25
Identity	ESI-MS	12280 ± 3 Da	Pass	12280	Pass	12280	Pass	12281	Pass	12281	Pass	12281	Pass	12281	Pass	12281
Purity	Reducing SDS-PAGE	≥ 95% EPICERTIN	Pass	100.00%	Pass	100.00%	Pass	100.00%	Pass	100.00%	Pass	100.00%	Pass	100.00%	Pass	100.00%
Purity	Size exclusion HPLC	16.4 ± 0.2 min retention time and ≥ 95% AUC	Pass	100.00%	Pass	100.00%	Pass	100.00%	Pass	100.00%	Pass	100.00%	Pass	100.00%	Pass	97.51%
Potency	GM1/KDEL ELISA	EC50 shift < ± 30% of reference standard	Pass	+2.85%	Pass	+2.56%	Pass	+8.53%	Pass	-1.7%	Pass	-5.3%	Fail	-38.8%	Pass	-26.1%

Table S3. EPICERTIN DS forced degradation stability assessment results summary: high temperature condition.

			High Temperature																	
**Test Parameter**	**Test Method**	**Acceptance Criteria**	**40 °C 24 hour**		**40 °C 48 hour**		**40 °C 72 hour**		**50 °C 24 hour**		**50 °C 48 hour**		**50 °C 72 hour**		**60 °C 24 hour**		**60 °C 48 hour**		**60 °C 72 hour**	
Purity	Reducing SDS-PAGE	≥ 95% EPICERTIN	Pass	100.00%	Pass	100.00%	Pass	100.00%	Pass	100.00%	Pass	100.00%	Pass	100.00%	Pass	100.00%	Pass	100.00%	Pass	100.00%
Purity	Size exclusion HPLC	16.4 ± 0.2 min retention time and ≥ 95% AUC	Pass	96.13%	Fail	68.32%	Fail	72.91%	Fail	58.23%	Fail	39.63%	Fail	64.96%	Fail	77.00%	Fail	74.25%	Fail	93.03%
Potency	GM1/KDEL ELISA	EC50 shift < ± 30% of reference standard	Pass	-16.37%	Pass	0.75%	Pass	-2.12%	Pass	-9.93%	Pass	0.19%	Pass	-3.69%	Pass	6.36%	Pass	-6.74%	Pass	-6.59%

Table S4. EPICERTIN DS forced degradation stability assessment results summary: agitation and freeze thaw cycle conditions.

			Agitation				Freeze Thaw Cycles									
**Test Parameter**	**Test Method**	**Acceptance Criteria**	**24 hour**		**48 hour**		**1**		**2**		**3**		**4**		**5**	
Purity	Reducing SDS-PAGE	≥ 95% EPICERTIN	Pass	100.00%	Pass	100.00%	Pass	100.00%	Pass	100.00%	Pass	100.00%	Pass	100.00%	Pass	100.00%
Purity	Size exclusion HPLC	16.4 ± 0.2 min retention time and ≥ 95% AUC	Pass	100.00%	Pass	100.00%	Pass	100.00%	Pass	100.00%	Pass	100.00%	Pass	100.00%	Pass	100.00%
Potency	GM1/KDEL ELISA	EC50 shift < ± 30% of reference standard	Pass	14.50%	Pass	5.20%	Pass	4.16%	Pass	3.60%	Pass	2.22%	Pass	9.90%	Pass	26.6%

Table S5. EPICERTIN DS forced degradation stability assessment results summary: pH and oxidation conditions.

			pH								Oxidation			
**Test Parameter**	**Test Method**	**Acceptance Criteria**	**1**		**4**		**7**		**10**		**0.2% 36 hour**		**2% 36 hour**	
Purity	Reducing SDS-PAGE	≥ 95% EPICERTIN	Pass	100.00%	Pass	100.00%	Pass	100.00%	Pass	100.00%	Pass	100.00%	Pass	100.00%
Purity	Size exclusion HPLC	16.4 ± 0.2 min retention time and ≥ 95% AUC	Fail	0.00%	Fail	0.00%	Pass	100.00%	Pass	100.00%	Pass	100.00%	Pass	100.00%
Potency	GM1/KDEL ELISA	EC50 shift < ± 30% of reference standard	Fail	4,128.50%	Fail	-335.92%	Pass	6.36%	Pass	10.54%	Pass	2.44%	Pass	-2.10%
Identity	ESI-MS	12280 ± 3 Da	N/A								Pass	12280	Fail	12297

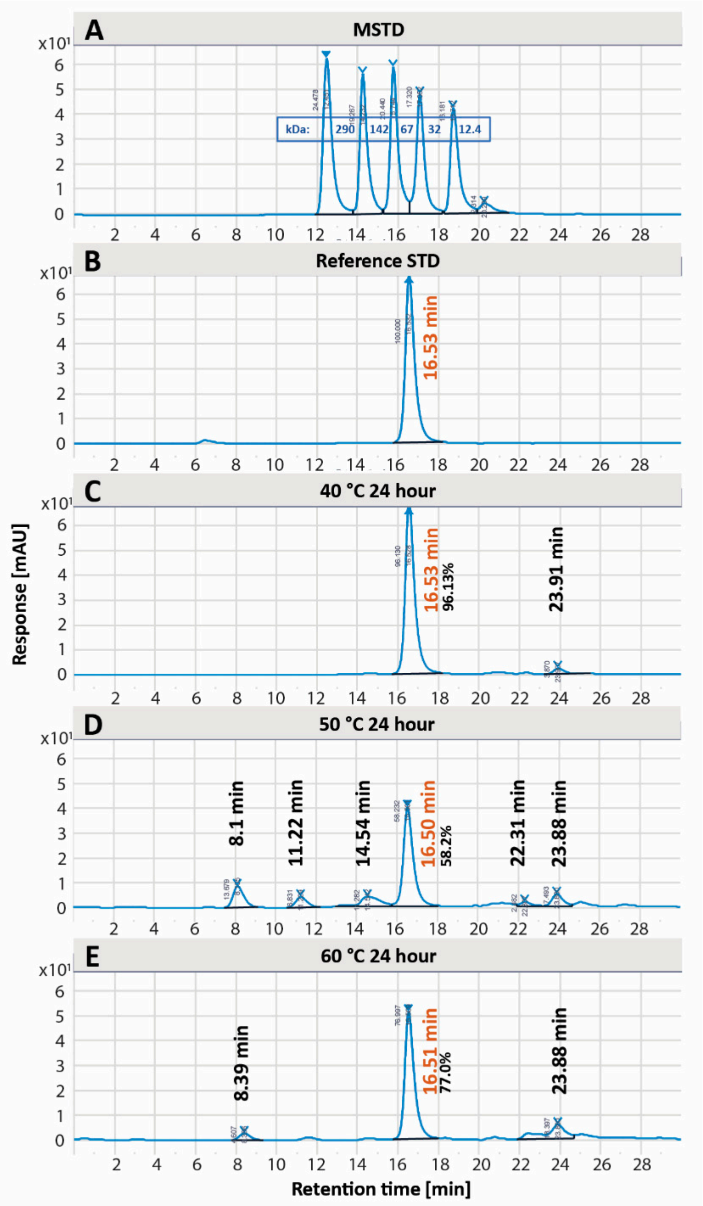

Figure S1. Forced degradation SEC-HPLC purity results for high temperature conditions at the 24 hour timepoint. A) Molecular weight standard. B) Reference standard. C) The 40 °C 24 hour condition was the only high temperature exposure that met the acceptance criteria, with a retention time meeting the specification of 16.4 ± 0.2 min and an AUC ≥ 95%. All other high temperature exposure conditions failed to meet the acceptance criteria, as highlighted by the chromatograms of the shortest exposure times (24 hours) of both 50 °C and 60 °C exposure temperatures (D and E, respectively).

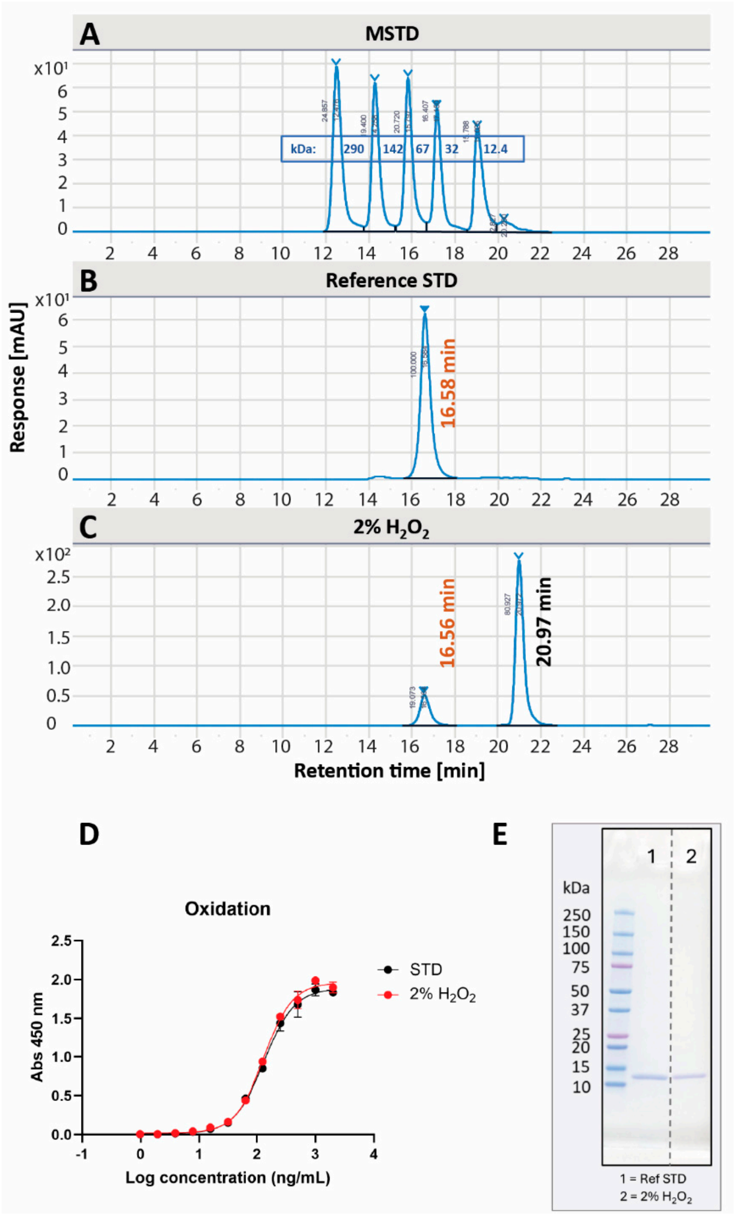

Figure S2. Forced degradation assessment of EPICERTIN DS 36 hour exposure to 2% H_2O_2. SEC-HPLC chromatograms of molecular weight standard (A) and reference standard (B). C) SEC-HPLC result showing oxidized EPICERTIN DS remains 100% pentamerized, with a retention time of 16.56 min. An additional peak is observed (retention time 20.97 min) which corresponds to the peak (by retention time and AUC) of 2% H_2O_2 in PBS alone. D) Representative GM1/KDEL ELISA of oxidized EPICERTIN DS, which met the specification of EC_{50} shift < ± 30% of the reference standard (EC_{50} shift of -2.10%). E) SDS-PAGE result of oxidized EPICERTIN DS is unchanged as compared to the reference standard.
